# Early pregnancy weight gain in pregnant women and the risk of gestational diabetes: a dose–response meta-analysis

**DOI:** 10.3389/fpubh.2026.1933184

**Published:** 2026-10-29

**Authors:** Jing Li, Mengyuan Yang, Yaxin Chen, Xin Qi, Lei Wu, Mei Wang, Yanhong Wang

**Affiliations:** School of Nursing, Lanzhou University, Lanzhou, China

**Keywords:** dose–response relationship, first trimester, gestational diabetes, meta-analysis, weight gain

## Abstract

**Background:**

Gestational diabetes mellitus is the most common metabolic disorder of pregnancy, and its global prevalence continues to rise. Although excessive gestational weight gain has been linked to an increased risk of gestational diabetes mellitus, the precise dose–response relationship during the early pregnancy remains unclear. This study aimed to systematically evaluate the association between early pregnancy weight gain (defined as first-trimester weight gain, approximately 12–13 weeks) and the risk of developing gestational diabetes mellitus and to quantify the dose–response relationship.

**Methods:**

A systematic search of PubMed, Embase, Web of Science, the Cochrane Library, China Biology Medicine disk, VIP Database for Chinese Technical Periodicals, WanFang Data, and China National Knowledge Infrastructure was conducted from database inception to December 2025 to identify studies examining the association between first-trimester weight gain and the risk of gestational diabetes mellitus. Two reviewers independently performed literature screening, data extraction, and risk of bias assessment. A traditional meta-analysis was performed using Stata 17.0 with a random-effects model using restricted maximum likelihood (REML) and Knapp–Hartung adjustment. The dose–response relationship was examined using restricted cubic spline regression and generalized least squares estimation. Subgroup analyses by GDM diagnostic criteria were performed.

**Results:**

Nine cohort studies, encompassing 66,673 participants and 13,130 incident cases of gestational diabetes mellitus, were included in the meta-analysis. In the primary analysis restricted to seven studies with strict first-trimester weight gain, insufficient weight gain was associated with a lower risk of GDM (RR 0.84, 95% CI 0.79–0.90), whereas excessive weight gain was associated with a 32% higher risk (RR 1.32, 95% CI 1.20–1.45). Each 1-kg increment in first-trimester weight gain was associated with a 19% increase in GDM risk (RR 1.19, 95% CI 1.15–1.23). No statistically significant departure from linearity was observed (*P* for non-linearity = 0.973). Subgroup analysis by GDM diagnostic criteria showed no significant between-subgroup difference for excessive weight gain (*p* = 0.810) and a borderline difference for insufficient weight gain (*p* = 0.082).

**Conclusion:**

Current evidence indicates that excessive first-trimester weight gain is associated with an increased risk of gestational diabetes mellitus, and no statistically significant departure from linearity was observed for the dose–response association between first-trimester weight gain and the risk of gestational diabetes mellitus. Weight monitoring and early intervention during the first trimester should be prioritized in clinical practice to reduce the risk of gestational diabetes mellitus.

**Systematic review registration:**

https://www.crd.york.ac.uk/PROSPERO/view/CRD420251140857.

## 1 Introduction

Gestational diabetes mellitus (GDM), defined as impaired glucose tolerance of varying severity with first onset or recognition during pregnancy (1), is the most common metabolic disorder of pregnancy. Concurrent with lifestyle transitions over recent decades, the rising prevalence of obesity among women of reproductive age and the increasing proportion of pregnancies at advanced maternal age have contributed to a sustained increase in the incidence of GDM. Globally, an estimated 14% of pregnancies are affected by this condition (2, 3). GDM not only elevates the risk of adverse perinatal outcomes, including cesarean section, gestational hypertension, macrosomia, and neonatal hypoglycaemia (4), but also carries profound long-term implications for both mother and child. Specifically, GDM substantially increases the lifetime risk of chronic non-communicable diseases, such as type 2 diabetes mellitus (T2DM) and cardiovascular disease (CVD), in both the mother and her offspring during adulthood (5–7). Early identification and management of modifiable risk factors for GDM are therefore essential to improving maternal and infant health.

Gestational weight gain (GWG) has been consistently identified as an independent risk factor for GDM (8, 9). Early pregnancy—a critical window for embryonic development and maternal metabolic adaptation—is thought to play a potentially decisive role in the onset and progression of GDM (10). Excessive weight gain during early pregnancy may contribute to heightened early insulin resistance, which in turn can lead to pancreatic β-cell exhaustion (11). This deterioration in β-cell function may impair the capacity to compensate for the physiological increase in insulin resistance as pregnancy advances, ultimately resulting in maternal hyperglycaemia and fetal overgrowth (11, 12). Although several original studies have examined the association between first-trimester weight gain and the risk of GDM, the evidence remains inconclusive. Some studies report that excessive first-trimester weight gain significantly increases GDM risk. Hedderson et al. (13) found in a multiracial nested case–control study that women in the highest category of first-trimester weight gain rate had an 80% elevated risk of GDM, and this association was independent of pre-pregnancy body mass index (BMI). Among women with normal pre-pregnancy BMI, a progressive upward trend in first-trimester weight gain was associated with a 23% increased risk of GDM (14). Other studies have also highlighted a significant association between first-trimester weight gain and GDM, noting that insufficient weight gain during this period may be associated with a reduced risk of GDM (15). Despite a growing body of research, the dose–response relationship between first-trimester weight gain and GDM risk remains unclear. Accordingly, this study aims to systematically evaluate the relationship between first-trimester weight gain and GDM risk and to employ a dose–response meta-analysis to further quantify this risk, thereby providing scientific evidence to inform clinical guidelines and public health strategies for weight management in early pregnancy.

## 2 Materials and methods

### 2.1 Design and registration

This study comprised a systematic review and dose–response meta-analysis of cohort studies. The review was conducted and reported in accordance with the Preferred Reporting Items for Systematic Reviews and Meta-Analyses (PRISMA) 2020 statement, and the completed PRISMA checklist is provided as Supplementary File 1. The systematic review was registered with PROSPERO (CRD420251140857).

### 2.2 Literature search strategies

A systematic search was conducted in PubMed, Embase, Web of Science, The Cochrane Library, CBM, VIP, WanFang Data, and CNKI from database inception to December 2025 to identify studies examining the association between weight change in early pregnancy and the risk of gestational diabetes. The search strategy combined subject headings and free-text terms, with adjustments tailored to the specific requirements of each database. To supplement the primary search, the reference lists of all included studies were also screened. English search terms included “Maternal Weight Gain,” “Weight Gain, Maternal,” “Pregnancy Weight Gain,” “Weight Gain, Pregnancy,” “Body Weight Change,” “Change, Body Weight,” “Gestational Diabetes Mellitus,” “Diabetes, Pregnancy-Induced,” and “Gestational Diabetes.” Chinese search terms encompassed “gestational diabetes,” “first trimester,” “weight gain,” and “pregnancy weight gain.”

### 2.3 Inclusion and exclusion criteria

#### 2.3.1 Inclusion criteria

Studies were eligible according to the PECOS framework:

P (Population): First-trimester pregnant women with singleton pregnancies, without GDM at baseline.

E (Exposure): First-trimester gestational weight gain (GWG; measured at approximately 12–13 weeks).

C (Comparator): Adequate GWG category (0.5–2 kg) as the reference.

O (Outcome): Gestational diabetes mellitus (GDM).

S (Study design): Cohort studies.

Studies were eligible if they reported GWG in at least three exposure categories, together with relative risk (RR), hazard ratio (HR), or odds ratio (OR) and corresponding 95% confidence intervals (CI) for each category, and provided extractable data on the number of cases and total population for each exposure level. Studies with broader exposure windows (e.g., weight gain up to 24–28 weeks) were excluded from the primary analysis and included only in sensitivity analyses.

#### 2.3.2 Exclusion criteria

Studies were excluded if they (1) were reviews, case reports, conference abstracts, or duplicate publications; (2) had incomplete data or lacked sufficient information to calculate the required effect sizes; (3) were published in languages other than Chinese or English; or (4) enrolled participants with pre-existing diabetes, chronic hypertension, renal disease, heart disease, or other severe chronic conditions that could affect glucose metabolism or pregnancy outcomes.

### 2.4 Literature screening and data extraction

Two reviewers independently performed the literature screening, data extraction, and cross-checking. Any disagreements were resolved through discussion or consultation with a third reviewer. During the screening process, titles were first examined; after excluding clearly irrelevant studies, the abstracts and full texts of potentially eligible studies were reviewed to determine final inclusion. Where necessary, the corresponding authors of original studies were contacted by email or telephone to obtain information that was unclear but essential for the analysis. Data extracted from each included study comprised: first author’s name, year of publication, country, total sample size, categories of first-trimester GWG, median weight gain within each category, number of GDM cases per category, total number of GDM cases, adjusted RR, and 95% CI. We also extracted information on the source of pre-pregnancy weight (self-reported vs. measured), the method of gestational weight measurement, the exact formula used for weight gain, and any reported validation of self-reported weight.

### 2.5 Assessment of risk of bias in included studies

Two reviewers independently assessed the risk of bias of the included studies, with disagreements resolved by discussion or consultation with a third reviewer. The 9-point Newcastle-Ottawa Scale (NOS) was used for risk of bias assessment (16). The scale evaluates three domains: selection of study groups, comparability between groups, and ascertainment of outcome. Based on total scores, studies were classified as low (0–3), moderate (4–6), or high (≥7) quality (17). In addition, we performed a GRADE assessment; because all studies were observational, the starting certainty was low, and the final certainty remained low after downgrading for risk of bias and inconsistency and upgrading for dose–response gradient.

### 2.6 Statistical analysis

All statistical analyses were performed using Stata 17.0. For the traditional meta-analysis, “appropriate weight gain” (0.5–2 kg) (18) was designated as the reference category, and pooled relative risks (RR) with 95% confidence intervals (CI) were calculated comparing “insufficient weight gain” (<0.5 kg) and “excessive weight gain” (>2 kg) against the reference. A random-effects model with restricted maximum likelihood (REML) and Knapp–Hartung adjustment was used for the primary analysis. Heterogeneity across studies was assessed using the *Q* test and *I*^2^ statistic. Sensitivity analyses were conducted by sequentially excluding individual studies and by including studies with broader exposure windows. Publication bias was assessed using funnel plots, together with the Begg rank-correlation test and the Egger linear regression test.

For the dose–response meta-analysis, a restricted cubic spline regression model was fitted to examine the shape of the association between first-trimester weight gain and the risk of GDM (19). A *p*-value ≥ 0.05 indicated no statistically significant departure from linearity. Model parameters were estimated using generalized least squares (20). The exposure dose for each weight gain category was assigned as the midpoint of its upper and lower bounds. For the highest open-ended category, the dose was estimated by adding half the interval width of the adjacent category to its lower bound (21); for the lowest open-ended category, the dose was estimated by subtracting half the interval width of the adjacent category from its upper bound (22). All included studies used the IOM first-trimester reference range (0.5–2 kg) except Chen et al. (23), which used the Chinese Nutrition Society (CNS) standard: insufficient <0 kg, adequate 0–2 kg, excessive >2 kg. To harmonize with the IOM framework, Chen et al. (2, 23) was recategorized as follows: CNS insufficient (<0 kg) → IOM insufficient (<0.5 kg); CNS adequate (0–2 kg) → IOM adequate (0.5–2 kg); CNS excessive (>2 kg) → IOM excessive (>2 kg). The 0.5 kg cutoff was chosen because all other studies used the IOM range, in which 0.5 kg is the lower bound of adequate gain. This harmonization is an approximation because 0–0.5 kg is classified as adequate under the CNS standard but as insufficient under the IOM standard. For the dose–response analysis, uniform midpoint doses of 0.25, 1.25, and 2.75 kg were assigned to the insufficient, adequate, and excessive categories, respectively. The fitted dose–response curve was then plotted.

## 3 Results

### 3.1 Literature screening process and results

The initial search yielded a total of 15,088 records; after a series of screening steps, 9 studies were ultimately included (15, 23–30). Two studies [Park et al. (29) and Lautredou et al. (30)] had broader early-pregnancy weight gain windows and were excluded from the primary analysis, leaving 7 studies (62,780 participants, 12,499 GDM cases) for the primary analysis. The literature screening process and results are shown in Figure 1.

**Figure 1:**
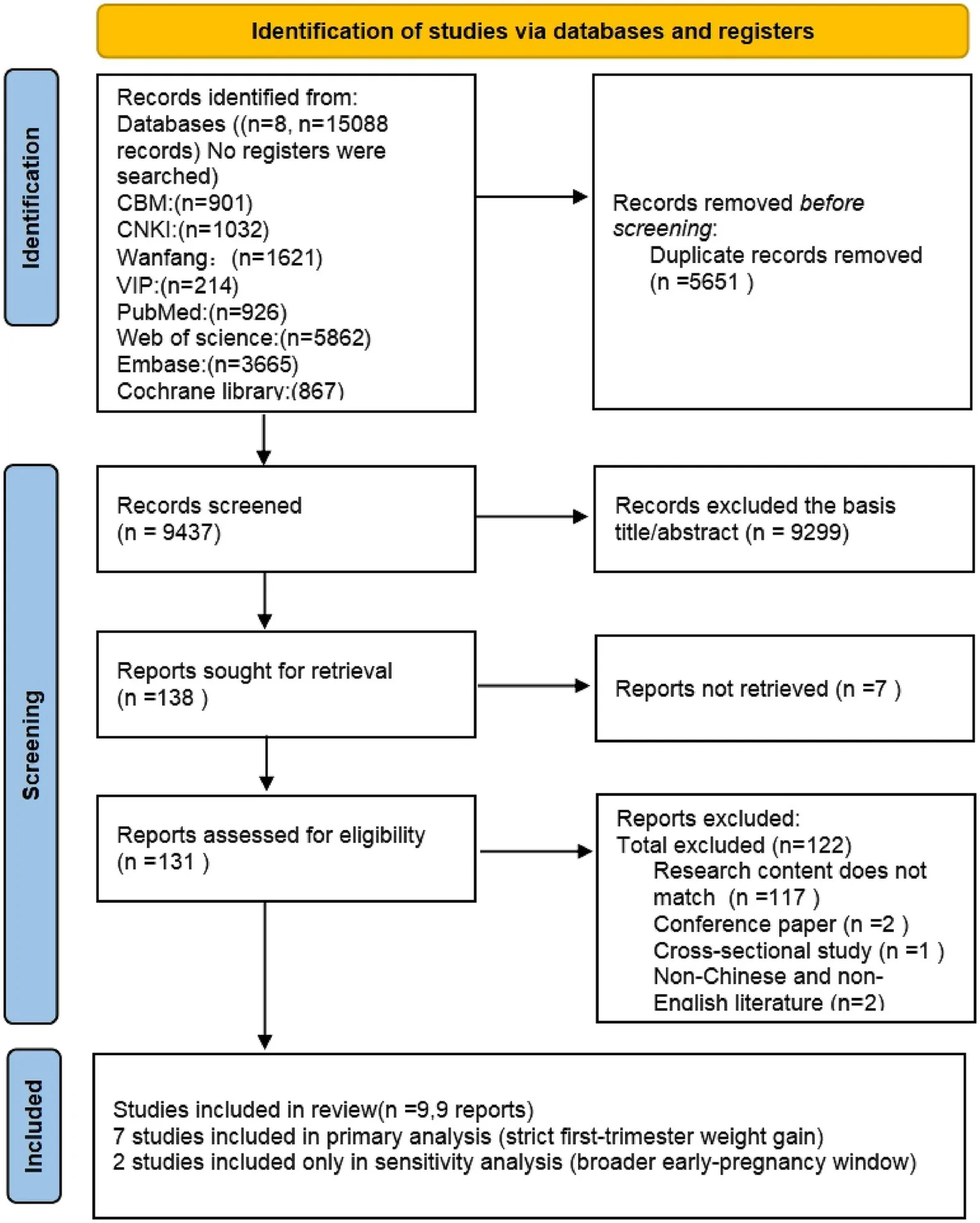
Literature screening process and results.

### 3.2 Basic characteristics and quality assessment of included studies

The 9 studies included a total of 66,673 participants, of whom 13,130 had gestational diabetes. All 9 studies received a NOS score of ≥7, indicating that the overall quality of the literature was high. Among the nine included cohorts, eight explicitly relied on self-reported pre-pregnancy weight, accounting for 63,511 of 66,673 participants (95.3%). Validation data were available in two cohorts: Zhong et al. (27) reported an intraclass correlation coefficient of 0.96, and Moore Simas et al. (28) reported *r* = 0.95 with no significant difference by BMI category as shown in Table 1.

**Table 1:** Basic characteristics of the study participants.

Study (year)	Country	Design	Exposure window	Total group	Exposure group	Median gwg	cases	Prevalence rate (%) per group	N total	Cases total	RRadj (95%CI)	Included in primary analysis
Liang et al. (2019) (24)	China	Prospective cohort	Pre-pregnancy to 12 wk	12,596	Insufficient: <0.5 kg	0.25	2,266	17.99	18,939	3,593	0.89(0.77 ~ 1.05)	Yes
				4,740	Adequate: 0.5 ~ 2 kg	1.25	933	19.68			1.00	
				1,603	Excessive: >2 kg	2.75	394	24.58			1.33(1.03 ~ 1.7)	
Dai et al. (2023) (25)	China	Prospective cohort	Pre-pregnancy to 12 wk	901	Insufficient: <0.5 kg	0.25	131	14.53	1,508	251	0.84(0.64 ~ 1.11)	Yes
				418	Adequate: 0.5 ~ 2 kg	1.25	72	17.22			1.00	
				189	Excessive: >2 kg	2.75	48	25.4			1.85(1.11 ~ 3.06)	
Dai et al. (2016) (26)	China	Prospective cohort	Pre-pregnancy to 12 ± 1 wk	302	Insufficient: <0.5 kg	0.25	48	15.89	682	116	1.23(0.63 ~ 2.38)	Yes
				208	Adequate: 0.5 ~ 2 kg	1.25	30	14.42			1.00	
				172	Excessive: >2 kg	2.75	38	22.09			2.2(1.12 ~ 4.35)	
Yin et al. (2022) (15)	China	Prospective cohort	Pre-pregnancy to 12 ± 1 wk	8,709	Insufficient: <0.5 kg	0.25	1,315	15.1	21,121	3,710	0.858(0.786 ~ 0.937)	Yes
				6,567	Adequate: 0.5 ~ 2 kg	1.25	1,158	17.63			1.00	
				5,845	Excessive: >2 kg	2.75	1,237	21.16			1.201(1.097 ~ 1.316)	
Moore Simas et al. (2019) (28)	USA	Retrospective cohort	Pre-pregnancy to 13 wk	636	Insufficient: <0.5 kg	0.25	31	4.87	1,616	113	0.59(0.35 ~ 1.01)	Yes
				395	Adequate: 0.5 ~ 2 kg	1.25	30	7.59			1.00	
				585	Excessive: >2 kg	2.75	52	8.89			1.08(0.67 ~ 1.76)	
Zhong et al. (2017) (27)	China	Prospective cohort	Pre-pregnancy to 13 ± 2 wk	357	Insufficient: <0.5 kg	0.25	16	4.48	2090	173	0.59(0.33 ~ 1.05)	Yes
				812	Adequate: 0.5 ~ 2 kg	1.25	58	7.14			1.00	
				921	Excessive: >2 kg	2.75	99	10.75			1.4(0.98 ~ 1.98)	
Chen et al. (2025) (23)	China	Retrospective cohort	Pre-pregnancy to 13 wk	4,002	Insufficient:<0.5 kg (CNS: <0 kg)	0.25	982	24.54	16,824	4,576	0.76(0.69 ~ 0.84)	Yes
				6,464	Adequate: 0.5 ~ 2 kg (CNS: 0–2 kg)	1.25	1721	26.62			1.00	
				6,358	Excessive: >2 kg (CNS: >2 kg)	2.75	1873	29.46			1.37(1.26 ~ 1.49)	
Lautredou et al. (2022) (30)	France	Prospective cohort	First visit to last pregnancy visit (max 1 mo before birth)	968	Insufficient: <0.5 kg	0.25	139	14.36	3,162	400	1.59(1.19 ~ 2.13)	No (sensitivity only)
				1,038	Adequate: 0.5 ~ 2 kg	1.25	103	9.92			1.00	
				1,156	Excessive: >2 kg	2.75	158	13.67			1.55(1.17 ~ 2.06)	
Park et al. (2014) (29)	Korea	Prospective cohort	Pre-pregnancy to 24–28 wk	108	Insufficient: <0.5 kg	0.25	22	20.37	731	198	0.437(0.239 ~ 0.8)	No (sensitivity only)
				165	Adequate: 0.5 ~ 2 kg	1.25	49	29.70			1.00	
				458	Excessive:>2 kg	2.75	127	27.73			0.779(0.522 ~ 1.162)	

*N* total, total number of participants enrolled in the study; Cases total, total number of incident GDM cases; Total group, total number of participants included in the dose–response analysis (sum across the three exposure categories); Exposure group, category of first‑trimester gestational weight gain; Median GWG, assigned median gestational weight gain (kg); Cases, number of GDM cases per exposure group; Prevalence rate per group, proportion of GDM within each exposure group (%); RR_adj, adjusted relative risk; CI, confidence interval relative risk.

Exposure groups: Insufficient, <0.5 kg; adequate, 0.5–2.0 kg; excessive, >2.0 kg, according to the IOM 2009 first-trimester reference range. For Chen et al. (2, 23), the Chinese Nutrition Society standard was used (adequate 0–2.0 kg; insufficient <0 kg; excessive >2.0 kg).

Median GWG: Midpoint dose assigned to each exposure category for the dose–response analysis: 0.25 kg (insufficient), 1.25 kg (adequate), and 2.75 kg (excessive).

RRadj: ORs converted to RRs using RR = OR/[1 − P₀ + (P₀ × OR)]; reference group = 1.00.

Included in primary analysis: Yes = strict first-trimester weight gain; No (sensitivity) = broader exposure window, sensitivity analysis only.

Park et al. (29) and Lautredou et al. (30) were not included in the primary analysis because their exposure windows were broader than the first trimester. They were included only in sensitivity analyses.

Chen et al. (2, 23) used the CNS standard (<0 kg, 0–2 kg, >2 kg), which was harmonized to <0.5 kg, 0.5–2 kg, and >2 kg for this meta-analysis. A sensitivity analysis excluding Chen et al. (2, 23) yielded consistent results (see Supplementary Figures S1, S2).

The covariates adjusted for in each original study are summarized in Supplementary Table S1. All studies adjusted for maternal age. Pre-pregnancy BMI was adjusted for in four studies (Liang Y, Dai Z, Y, Zhong C, Chen). Other commonly adjusted covariates included parity, family history of diabetes, ethnicity, education, smoking, and gestational age at weight measurement. However, the adjustment sets differed across studies.

### 3.3 Results of the meta-analysis

#### 3.3.1 Insufficient and excessive weight gain in early pregnancy and the risk of gestational diabetes mellitus (GDM)

##### 3.3.1.1 Insufficient first-trimester weight gain and risk of GDM

The primary analysis included seven cohort studies with clearly defined first-trimester weight measurement windows. Low heterogeneity was observed across studies (*τ*^2^ = 0.00; *I^2^* = 1.91%; *H*^2^ = 1.02; *Q* = 6.97, df = 6, *p* = 0.32), and a random-effects model with restricted maximum likelihood (REML) was used to estimate the pooled effect. Compared with women who had appropriate weight gain, those with insufficient first-trimester weight gain had a lower risk of GDM (pooled RR = 0.84, 95% CI 0.80–0.89; z = −6.41, *p* < 0.001; Figure 2), corresponding to a 16% relative risk reduction (95% CI: 11–20%).

**Figure 2:**
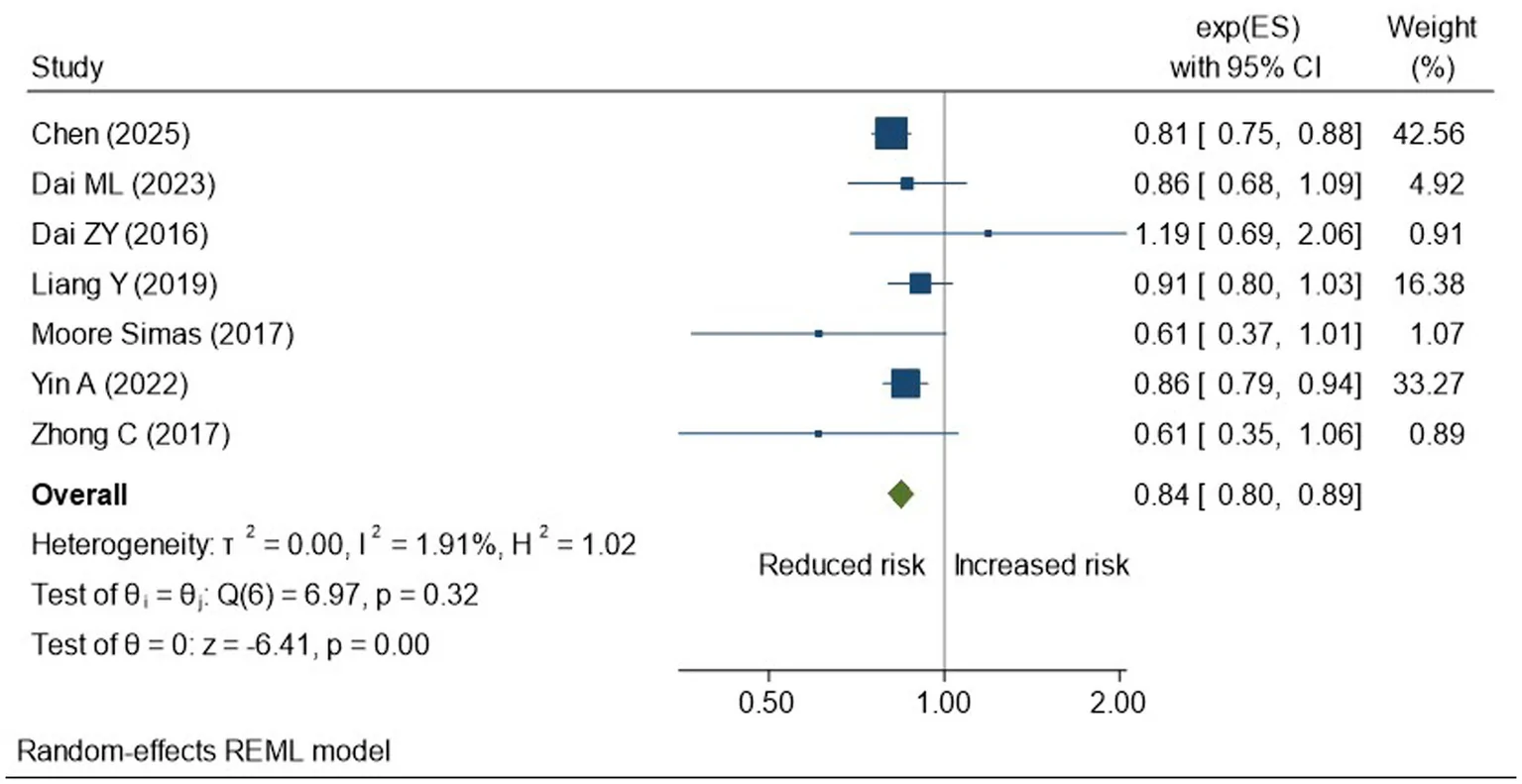
Forest plot showing the association between insufficient weight gain and the risk of developing gestational diabetes.

##### 3.3.1.2 Excessive first-trimester weight gain and risk of GDM

For excessive first-trimester weight gain, seven studies were included in the primary analysis. Low-to-moderate heterogeneity was observed (*τ*^2^ = 0.00; *I^2^* = 32.14%; *H*^2^ = 1.47; *Q* = 9.35, df = 6, *p* = 0.15), and a random-effects REML model was applied. Compared with appropriate weight gain, excessive first-trimester weight gain was associated with a higher risk of GDM (pooled RR = 1.32, 95% CI 1.20–1.45; *z* = 5.78, *p* < 0.001; Figure 3), corresponding to a 32% relative risk increase (95% CI: 20–45%).

**Figure 3:**
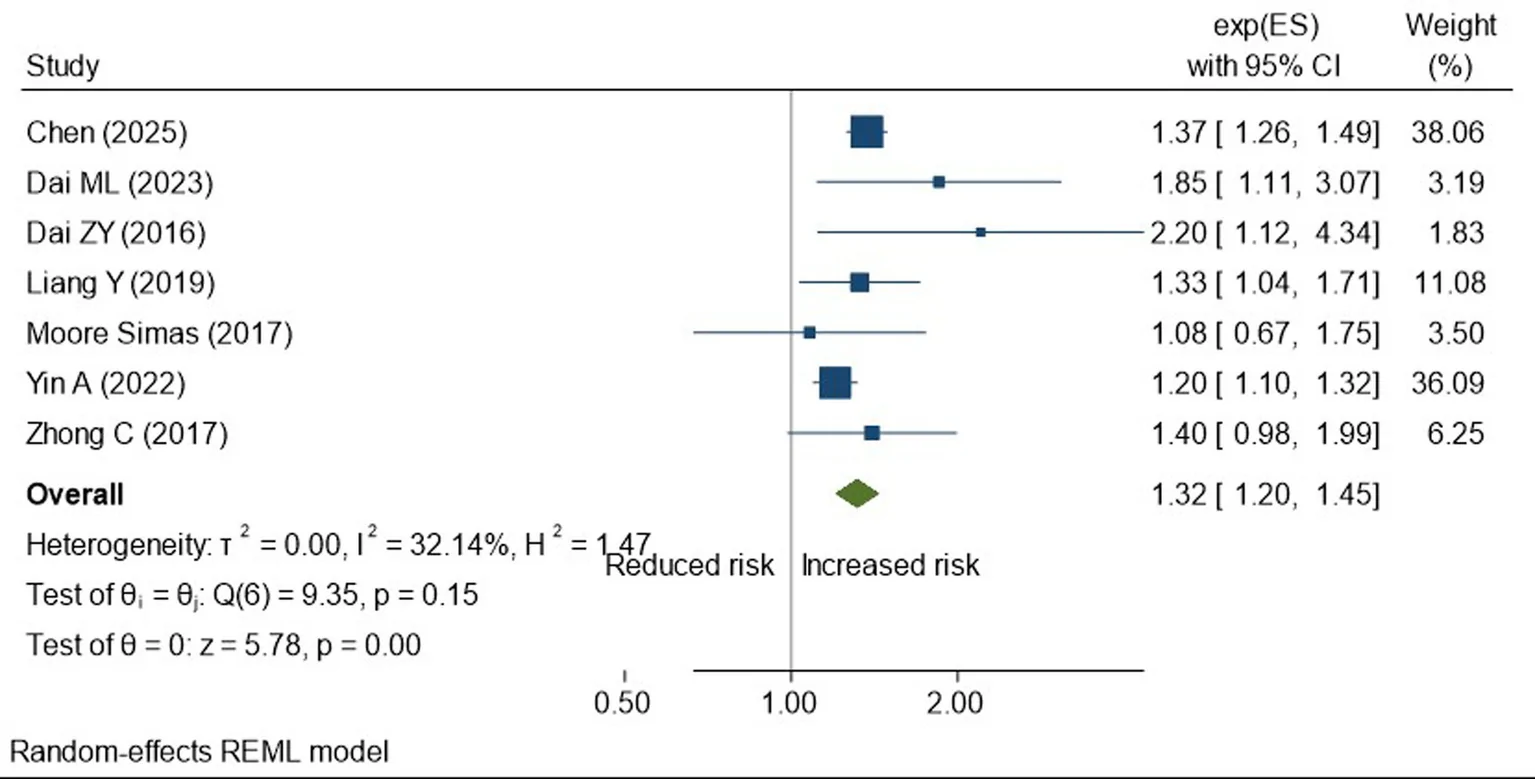
Forest plot showing the association between excessive weight gain and the risk of developing GDM.

In sensitivity analyses excluding Chen et al. (2, 23) using a random-effects model with restricted maximum likelihood and Knapp–Hartung adjustment, the pooled estimates were consistent with the primary analysis for both insufficient weight gain (RR 0.87, 95% CI 0.81–0.93; *I^2^* = 0.00%) and excessive weight gain (RR 1.29, 95% CI 1.14–1.46; *I^2^* = 18.59%) (Supplementary Figures S1, S2).

#### 3.3.2 Publication bias and sensitivity analysis

##### 3.3.2.1 Insufficient weight gain

Publication bias and sensitivity analyses for insufficient first-trimester weight gain are presented in Figure 4. The funnel plot showed a roughly symmetrical distribution of studies around the pooled estimate (Figure 4A). The Egger test indicated no statistically detectable small-study effects (bias = −0.145, 95% CI − 2.083 to 1.793, *p* = 0.855); however, given the limited number of included studies (*n* = 7), this finding should be interpreted as an absence of detectable small-study effects rather than definitive evidence of no publication bias. In the leave-one-out sensitivity analysis, the pooled RR ranged from 0.82 to 0.86 after sequential exclusion of each study, and all 95% CIs remained below 1.00 (Figure 4B). The direction and statistical significance of the pooled estimate remained unchanged, indicating that the association between insufficient first-trimester weight gain and lower GDM risk was robust and not driven by any single study.

**Figure 4:**
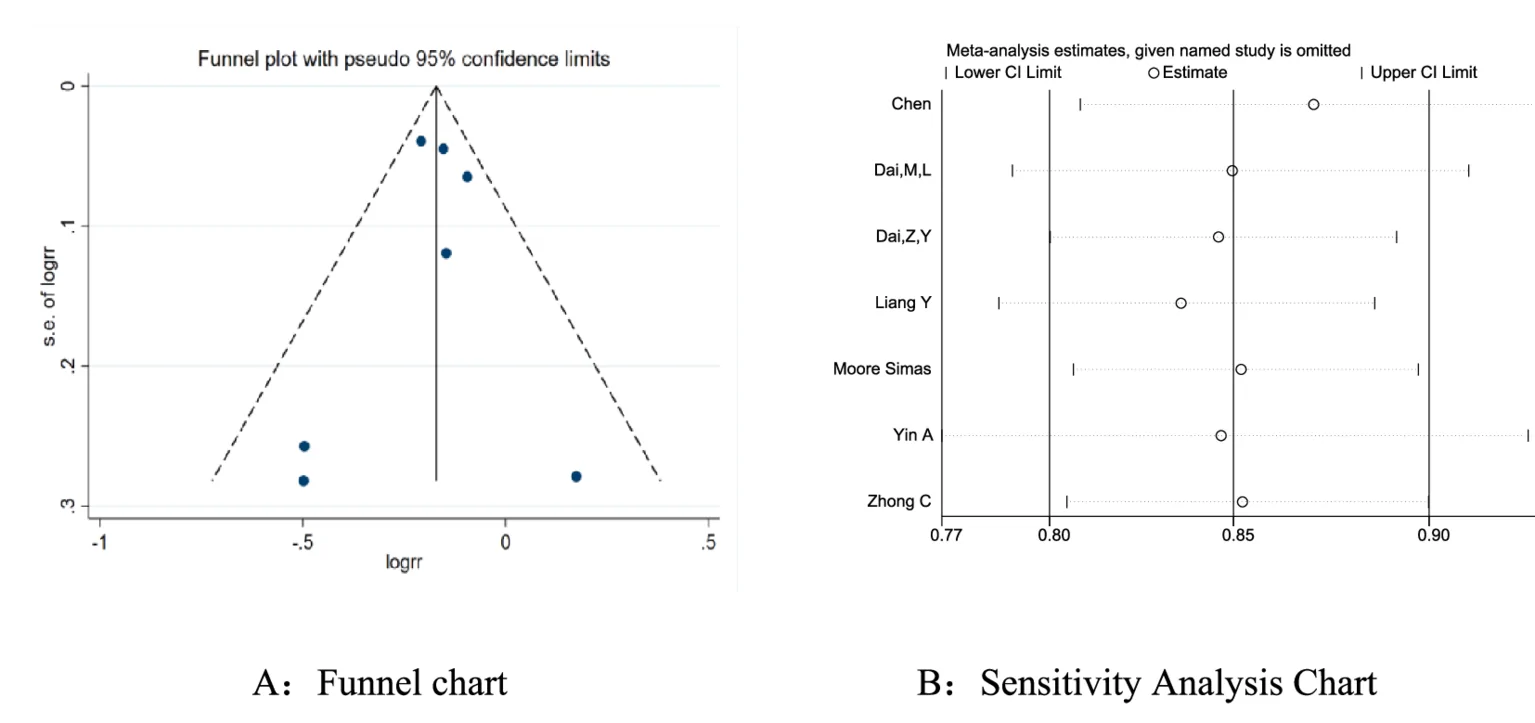
Publication bias in the group with insufficient weight gain.

##### 3.3.2.2 Excessive weight gain

Publication bias and sensitivity analyses for excessive first-trimester weight gain are presented in Figure 5. The funnel plot showed a roughly symmetrical distribution of studies around the pooled estimate (Figure 5A). The Egger test indicated no statistically detectable small-study effects (bias = 0.748, 95% CI − 1.161 to 2.657, *p* = 0.360); however, given the limited number of included studies (*n* = 7), this finding should be interpreted as an absence of detectable small-study effects rather than definitive evidence of no publication bias. In the leave-one-out sensitivity analysis, the pooled RR ranged from 1.31 to 1.34 after sequential exclusion of each study, and all 95% CIs remained above 1.00 (Figure 5B). The direction and statistical significance of the pooled estimate remained unchanged, indicating that the association between excessive first-trimester weight gain and higher GDM risk was robust and not driven by any single study.

**Figure 5:**
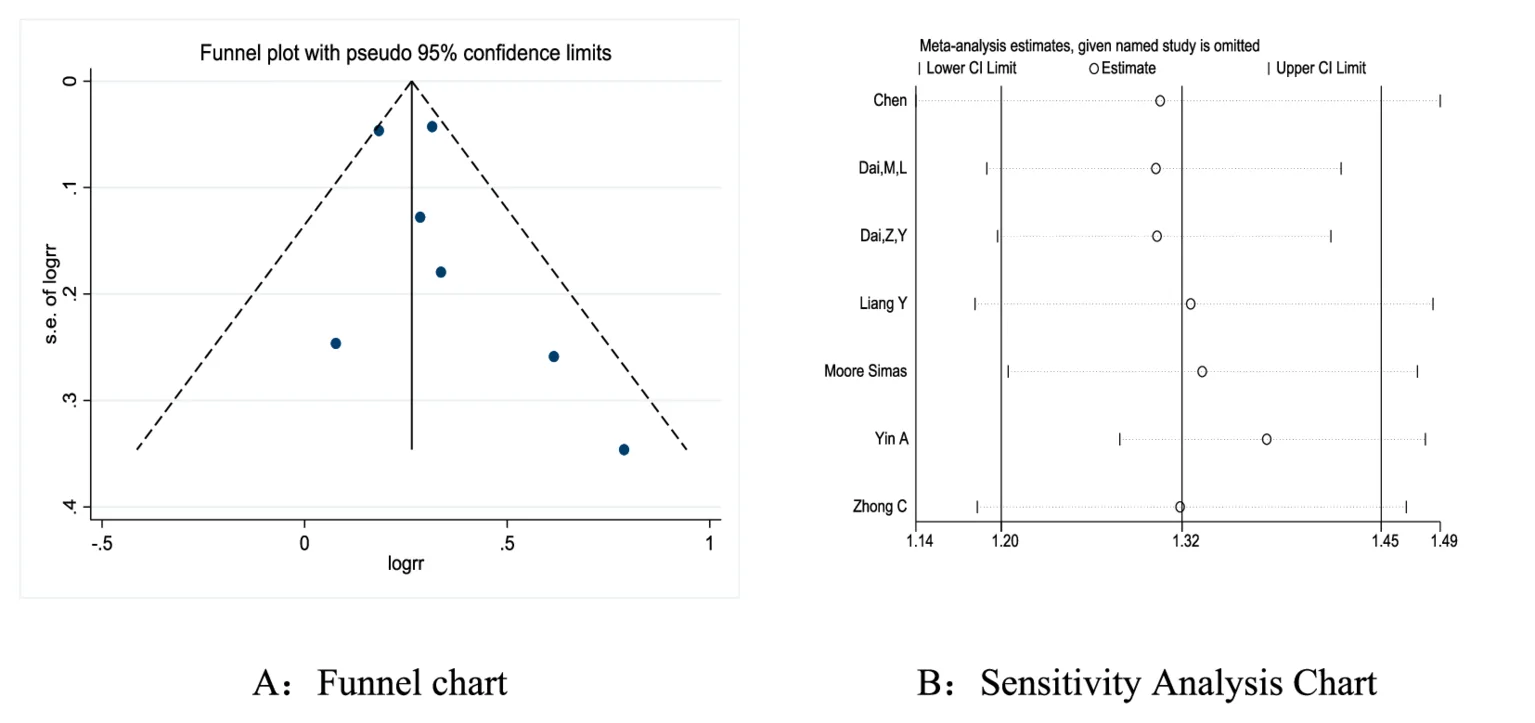
Publication bias in the group with excessive weight gain.

##### 3.3.2.3 Sensitivity analysis including broader-window studies

In sensitivity analyses that included two additional studies with broader exposure windows [Park et al. (29) and Lautredou et al. (30)], the pooled RR for excessive weight gain was 1.31 (95% CI 1.18–1.45), consistent with the primary analysis. For insufficient weight gain, heterogeneity increased substantially (*I^2^* = 88.8%), and the association was no longer statistically significant (RR 0.87, 95% CI 0.72–1.04, *p* = 0.13), largely attributable to Lautredou et al. (30). These findings support restricting the primary analysis to strict first-trimester weight gain (Supplementary Figures S3, S4).

#### 3.3.3 Subgroup analysis

Subgroup analyses were performed according to GDM diagnostic criteria (ADA vs. IADPSG/WHO). For insufficient first-trimester weight gain, the ADA subgroup included two studies and yielded a pooled RR of 0.61 (95% CI 0.60–0.62), whereas the IADPSG/WHO subgroup included five studies and yielded a pooled RR of 0.85 (95% CI 0.79–0.92). The test for between-subgroup differences was borderline non-significant (Q_b = 3.03, df = 1, *p* = 0.082). For excessive first-trimester weight gain, the ADA subgroup included two studies and yielded a pooled RR of 1.28 (95% CI 0.27–6.15), while the IADPSG/WHO subgroup included five studies and yielded a pooled RR of 1.33 (95% CI 1.12–1.58). The between-subgroup difference was not statistically significant (Q_b = 0.06, df = 1, *p* = 0.810). Given that the ADA subgroup contained only two studies, these results should be interpreted as exploratory (Table 2).

**Table 2:** Subgroup analysis of the association between weight gain and risk of gestational diabetes mellitus by GDM diagnostic criteria.

Outcome	Subgroup	No. of studies	Pooled RR (95% CI)	*I*^2^ (%)	*P* (within)	*P* (between)
Insufficient	ADA	2	0.61 (0.60–0.62)	0	0.996	0.082
	IADPSG/WHO	5	0.85 (0.79–0.92)	7.88	0.41	–
	Overall	7	0.84 (0.80–0.89)	1.91	0.324	–
Excessive	ADA	2	1.28 (0.27–6.15)	0	0.395	0.81
	IADPSG/WHO	5	1.33 (1.12–1.58)	47.22	0.072	–
	Overall	7	1.32 (1.20–1.45)	32.14	0.155	–

#### 3.3.4 Dose–response meta-analysis

A total of seven cohort studies with clearly defined first-trimester weight measurement windows were included in the dose–response meta-analysis. A restricted cubic spline regression model was fitted to the data. The non-linearity test indicated no significant departure from linearity in the relationship between first-trimester weight gain and the risk of GDM (*p* > 0.05). A linear dose–response model was then fitted using random-effects meta-regression with restricted maximum likelihood (REML) and the Knapp-Hartung adjustment. The reference point was set at 1.25 kg, corresponding to the midpoint of the 0.5–2 kg appropriate weight gain category (Figure 6). The model showed a positive linear trend: each 1 kg increment in first-trimester weight gain was associated with a 19% increase in the risk of GDM (RR = 1.19, 95% CI 1.15–1.23; t = 11.11, *p* < 0.001).

**Figure 6:**
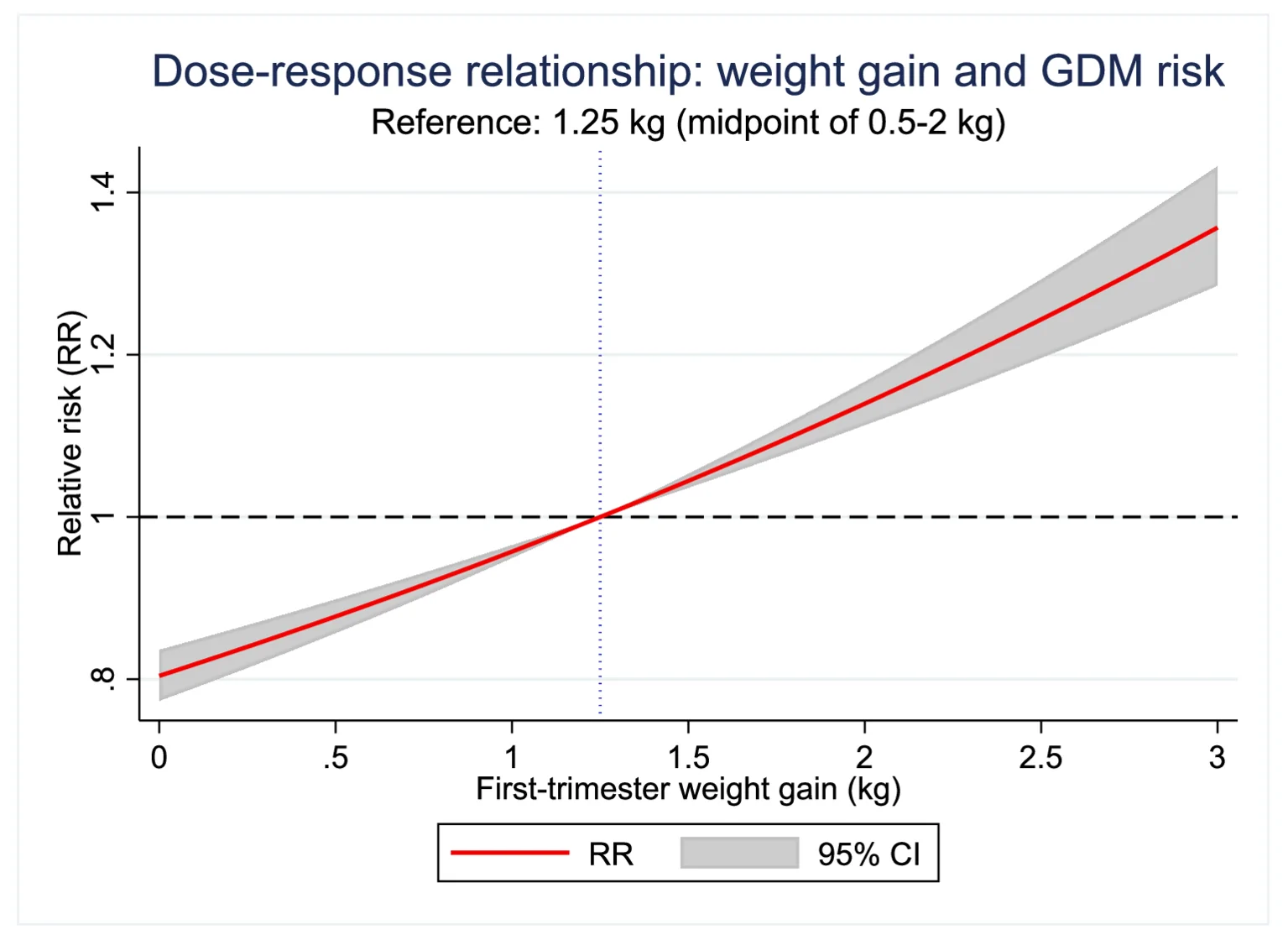
Dose–response relationship between weight gain and the risk of developing GDM.

In the dose–response sensitivity analysis excluding Chen et al. (23), each 1-kg increment in first-trimester weight gain was associated with RR 1.16 (95% CI 1.10–1.21), consistent with the primary analysis (RR 1.19, 95% CI 1.15–1.23) (Supplementary Figure S5).

#### 3.3.5 GRADE assessment

The certainty of evidence was low for both outcomes. Starting from low for observational studies, we downgraded for risk of bias (self-reported pre-pregnancy weight) and inconsistency (in sensitivity analyses). We upgraded for dose–response gradient. The final certainty remained low (Table 3).

**Table 3:** The grading of recommendations assessment, development and evaluation (GRADE) assessment.

Outcome	No. of studies	Design	Risk of bias	Inconsistency	Indirectness	Imprecision	Publication bias	Upgrade	Certainty	Effect size (95% CI)
Excessive GWG vs. adequate	7	Cohort	Serious^1^	Not serious (*I*^2^ = 32.14%)	Not serious	Not serious	Undetected	Dose–response^3^	Low	RR 1.32 (1.20–1.45)
Insufficient GWG vs. adequate	7	Cohort	Serious^1^	Not serious in primary (*I*^2^ = 1.91%); serious in sensitivity (*I*^2^ = 88.8%)^2^	Not serious	Not serious	Undetected	Dose–response	Low	RR 0.84 (0.80–0.89)

^1^Self-reported pre-pregnancy weight, retrospective designs. ^2^Sensitivity analysis including broader-window studies showed high heterogeneity. ^3^Dose–response gradient upgrades certainty by one level, but final certainty remains low.

## 4 Discussion

This dose–response meta-analysis examined the association between first-trimester gestational weight gain and the risk of GDM. To our knowledge, it is the first to comprehensively quantify the dose–response relationship between these two factors using a dose–response meta-analysis. Results from the random-effects model showed that, compared with appropriate weight gain, insufficient first-trimester weight gain was associated with an approximately 16% lower risk of GDM, whereas excessive weight gain was associated with an approximately 32% higher risk. The dose–response analysis revealed a positive linear trend, with no statistically significant departure from linearity, and each 1 kg increment in first-trimester weight gain was associated with a 19% increase in GDM risk (RR 1.19, 95% CI 1.15–1.23). These findings support early monitoring of first-trimester weight gain and highlight the need for prospective intervention studies to determine whether modifying early weight gain can reduce GDM risk.

### 4.1 Dose–response relationship between first-trimester weight gain and the risk of gestational diabetes

Previous studies examining the association between gestational weight gain and GDM have primarily focused on the second and third trimesters or on total pregnancy weight gain, with comparatively limited attention directed toward the critical window of the first trimester. Drawing on pooled estimates from cohort studies, the present findings clearly indicate that the magnitude of weight gain is significantly associated with GDM risk as early as the first trimester. To our knowledge, this study is the first to employ a dose–response meta-analysis to comprehensively quantify the relationship between first-trimester weight gain and GDM risk, revealing a positive, graded association; no statistically significant departure from linearity was observed. Specifically, each 1 kg increment in first-trimester weight gain was associated with an19% increase in the risk of GDM. This finding provides a more precise quantitative benchmark for weight management in early pregnancy and underscores the necessity of weight control during this critical period.

The results of the traditional meta-analysis are largely consistent with evidence from several prospective cohort studies. A substantial body of research has demonstrated that excessive first-trimester weight gain is associated with an elevated risk of GDM. MacDonald et al. (14) reported that weight gain exceeding expected levels during the first trimester increased GDM risk. Similarly, a prospective study conducted by Lan et al. (31) in southwestern China showed that first-trimester weight gain surpassing the IOM-recommended range was significantly associated with a higher risk of GDM. The association between rapid weight gain in early pregnancy and GDM risk has been further corroborated by multiple investigations. One study found that, after adjustment for potential confounders, women with rapid early pregnancy weight gain had a 1.92-fold higher risk of GDM compared with those who gained weight appropriately (13). Hedderson et al. (13) also highlighted that rapid weight gain in early pregnancy, particularly during the first trimester, may increase a woman’s risk of GDM, and that this association is more pronounced among overweight and non-white women. Collectively, these lines of evidence, derived from diverse racial and ethnic populations and employing varying weight gain assessment methods, lend support to the conclusions of the present study. Furthermore, our finding that insufficient first-trimester weight gain was associated with a reduced risk of GDM aligns with the conclusions of Chen et al. (23). That study indicated that insufficient first-trimester weight gain could lower GDM risk, especially among young women, those with elevated fasting blood glucose, and those with low high-density lipoprotein (HDL) or triglyceride levels, without adversely affecting neonatal birth weight. However, this observational association should not be interpreted as evidence that intentional caloric restriction or weight loss prevents GDM. Insufficient gestational weight gain has been associated with increased risks of small-for-gestational-age infants and preterm birth; clinical decisions should balance GDM risk against fetal growth outcomes conflicting results have been reported by Sun et al. (32) and Lewandowska et al. (33), who observed that insufficient weight gain was associated with an increased risk of GDM. Such discrepancies may be attributable to differences in genetic background, dietary patterns, and GDM diagnostic criteria across study populations; additionally, inconsistent definitions of “insufficient weight gain” may have contributed to the divergent findings.

### 4.2 Subgroup analysis and discussion of sources of heterogeneity

We did not conduct a formal subgroup analysis stratified by country because most country-level subgroups comprised only a single study, and single-study subgroups are unable to yield robust meta-analytic estimates. Country may also serve as a proxy for multiple other differences, including ethnicity, pre-pregnancy BMI distribution, dietary patterns, GDM diagnostic criteria, healthcare systems, and exposure definitions. We therefore focused on GDM diagnostic criteria as a more clinically meaningful and interpretable source of heterogeneity, while acknowledging that country-level differences may still contribute to between-study heterogeneity.

Subgroup analysis stratified by GDM diagnostic criteria showed no significant between-subgroup difference in the association between excessive weight gain and GDM risk when comparing the IADPSG/WHO/China criteria subgroup with the ADA criteria subgroup (*p* = 0.810). In contrast, the between-subgroup difference for insufficient weight gain was borderline (*p* = 0.082), with a stronger association observed in the ADA subgroup (RR = 0.61 vs. 0.85). This discrepancy may be attributable to differences in diagnostic sensitivity: the IADPSG/WHO criteria use a 75-g oral glucose tolerance test with a fasting glucose threshold of 5.1 mmol/L and require only one abnormal value for diagnosis, resulting in higher sensitivity and the potential detection of more mild GDM cases, thereby diluting the strength of the association; whereas the ADA criteria may use a one-step or two-step approach with different thresholds, potentially identifying more severe GDM cases. However, the ADA subgroup included only two studies; these results should therefore be interpreted with caution and considered exploratory.

In sensitivity analyses that included two studies with broader exposure windows [Park et al. (29) and Lautredou et al. (30)], the association between excessive weight gain and GDM remained robust (RR = 1.31, 95% CI 1.18–1.45), whereas heterogeneity increased substantially in the insufficient weight gain group (*I^2^* = 88.8%) and the association was no longer statistically significant (RR = 0.87, 95% CI 0.72–1.04, *p* = 0.13). Lautredou et al. (27) assessed total weight gain from the first visit to late pregnancy, which includes weight changes occurring after GDM diagnosis and thus carries a risk of reverse causation. This further supports restricting the primary analysis to studies with strict first-trimester weight gain assessment.

### 4.3 Discussion of potential mechanisms

Growing evidence has elucidated the biological mechanisms through which excessive weight gain in early pregnancy elevates the risk of gestational diabetes mellitus (GDM). First, early pregnancy constitutes a critical window for maternal metabolic adaptation. Compared with the non-pregnant state, insulin sensitivity declines by approximately 50–60% during pregnancy, with this physiological insulin resistance reaching its peak in late gestation (34). Excessive weight gain in early pregnancy can precipitate premature and disproportionate accumulation of visceral adipose tissue. The resultant increase in free fatty acid release from adipose depots interferes with insulin signaling in skeletal muscle via the Randle cycle (glucose–fatty acid cycle), thereby further exacerbating insulin resistance (35). Second, as an active endocrine organ, expanded adipose tissue secretes elevated levels of pro-inflammatory cytokines (e.g., TNF-α, IL-6) and adipokines such as leptin and resistin, while the production of the insulin-sensitizing adipokine adiponectin is concurrently suppressed. This pro-inflammatory/anti-inflammatory imbalance can directly impair pancreatic β-cell function and attenuate glucose uptake efficiency in peripheral tissues (36). Third, evidence from Van Raaij et al. (37) suggests that the proportion of maternal fat deposited during early pregnancy is disproportionately greater than that deposited in later pregnancy, and that the expansion of adipose tissue is inversely correlated with the decline in insulin sensitivity. This disproportionate early fat accretion may be a key reason why first-trimester weight gain exerts a more pronounced impact on GDM risk than weight gain in mid-to-late pregnancy.

## 5 Clinical and public health implications

The findings of this study suggest that excessive first-trimester weight gain is associated with an increased risk of GDM and that early pregnancy may represent a potential window for GDM prevention. Current guidelines, including the 2009 IOM recommendations and those issued by the Chinese Nutrition Society, provide overall gestational weight gain targets stratified by pre-pregnancy BMI but offer limited guidance on the rate of weight gain specifically during the first trimester. Our findings highlight the need for future randomized controlled trials to determine whether interventions aimed at regulating first-trimester weight gain can reduce GDM risk; such trials should also evaluate potential harms, including insufficient weight gain and its association with small-for-gestational-age infants and preterm birth. For women with insufficient first-trimester weight gain, although a reduced risk of GDM was observed, clinical decisions should be based on a comprehensive assessment incorporating fetal ultrasound indicators and pre-pregnancy nutritional status, and indiscriminate restriction of weight gain should be avoided. Future studies should perform dose–response analyses stratified by pre-pregnancy BMI and include diverse ethnic groups to identify BMI-specific thresholds and intervention targets.

## 6 Limitations

This study has the following limitations: The search was restricted to English- and Chinese-language databases, and pre-pregnancy weight was self-reported in most included studies, which may have introduced language and recall bias; measurement instruments also varied across studies. We were unable to perform analyses stratified by pre-pregnancy BMI or rate of weight gain because the original studies did not report these data, and adjustment sets differed across studies; residual confounding therefore cannot be excluded. As an observational meta-analysis, causal inference cannot be established, and the most of the included studies were from East Asia, and most were conducted in China. Two studies with broader exposure windows were included only in sensitivity analyses; clinical heterogeneity may persist despite low-to-moderate statistical heterogeneity. Finally, harmonizing Chen et al.’s (2, 23) CNS categories to the IOM framework is an approximation, as 0–0.5 kg is classified differently between the two standards; however, sensitivity analyses excluding Chen et al. (23) yielded consistent results for both categorical and dose–response analyses.

## 7 Conclusion

In summary, this study provides evidence of a positive dose–response association between first-trimester weight gain and the risk of developing GDM, with no statistically significant departure from linearity. Excessive weight gain was associated with an increased risk, whereas insufficient weight gain was associated with a reduced risk. However, this observational association should not be interpreted as supporting caloric restriction or intentional weight loss during pregnancy, given the established risks of small-for-gestational-age infants and preterm birth. These findings underscore the importance of prioritizing weight monitoring and management as early as the first trimester, and suggest that regulating the rate of weight gain during this critical period may contribute to lowering the risk of GDM and related adverse pregnancy outcomes. Future studies should perform dose–response analyses stratified by pre-pregnancy BMI and include diverse ethnic groups, as the current evidence is predominantly derived from East Asia. The methods used to measure gestational weight and pre-pregnancy weight varied across studies, which may introduce measurement heterogeneity. Despite low-to-moderate statistical heterogeneity, clinical heterogeneity may still exist owing to differences in study populations, exposure definitions, and diagnostic criteria.

## Data Availability

The original contributions presented in the study are included in the article/Supplementary material, further inquiries can be directed to the corresponding author.
