# Supplementary material for: Early pregnancy weight gain in pregnant women and the risk of gestational diabetes: a dose–response meta-analysis
Source: Front Public Health. 2026 Oct 29;14:1933184. doi: 10.3389/fpubh.2026.1933184 (PMC13754856; doi:10.3389/fpubh.2026.1933184)
Supplement: Supplementary file 1 [file Table_1.docx]

Table S1: Basic Characteristics of the Study Participants

| Study  (year) | Source of pre-pregnancy weight | GDM diagnostic criteria | Effect measure | Adjusted variables | Funding source | Conflicts of interest | quality |
| --- | --- | --- | --- | --- | --- | --- | --- |
| LiangY  (2019) | Self-reported | IADPSG | OR | Age at delivery, pre-pregnancy BMI | Not reported | Not reported | 7 |
| Dai ML  (2023) | Self-reported via questionnaire | WHO 2013 | OR | Age at delivery | Not reported | Not reported | 8 |
| Dai ZY  (2016) | Self-reported via questionnaire | Chinese guideline 2014 | OR | Age, pre-pregnancy BMI, family history of diabetes, family history of hypertension, exercise in first trimester, parity, education, household income, dietary energy | Chinese Nutrition Society（CNS-2012-002） | Not reported | 9 |
| Yin A  (2022) | Self-reported | Chinese Ministry of Health 2011 (IADPSG-based) | RR | Maternal age, ethnicity, education, employment, assisted reproduction, gravidity | National Natural Science Foundation of China (Nos. 81830041 and 81771611); Shenzhen Science and Technology Innovation Committee Special Funding for Future Industry (No. JCYJ20170412140326739) | None declared | 7 |
| Moore Simas  (2017) | Self-reported (medical record) | ADA criteria (50-g screen, 100-g OGTT) | OR | Age, study site | National Institutes of Health 5R01 DK064902; NIH grants KL2TR000160 and U01HL015268 (partial support for Dr. Waring) | The authors declare no competing interest | 6 |

Continued Table S1: Basic Characteristics of the Study Participants

| Study  (year) | Source of pre-pregnancy weight | GDM diagnostic criteria | Effect measure | Adjusted variables | Funding source | Conflicts of interest | quality |
| --- | --- | --- | --- | --- | --- | --- | --- |
| Zhong C  (2017) | Self-reported | ADA criteria (75-g OGTT) | OR | Maternal age, pre-pregnancy BMI, family history of diabetes, poor sleep quality in early pregnancy | National Program on Basic Research Project of China (No. 2013FY114200) | None declared | 8 |
| Chen  (2025) | Self-reported | Chinese Ministry of Health 2011 (IADPSG-based) | OR | Pre-pregnancy BMI, maternal age, parity, education, gestational age at delivery, fasting glucose, triglycerides, HDL, GWG | National Natural Science Foundation of China (Nos. 81830041 and 81771611); Shenzhen Science and Technology Innovation Committee Special Funding for Future Industry (No. JCYJ20170412140326739) | The authors declare no competing interests | 8 |
| Lautredou  (2022) | Not specified | Fasting blood glucose at 24–28 wk (criteria not detailed) | OR | Age, parity, geographical origin, tobacco use, gestational age, preexisting diabetes, preexisting chronic hypertension | Institutional support from Brest University Hospital; no specific grant | The authors declare no known competing financial interests or personal relationships | 7 |
| Park  (2014) | Self-reported | Carpenter–Coustan criteria (100-g OGTT) | OR | Maternal age, family history of diabetes, smoking, gestational weeks at GDM screening | Korea Health 21 R&D Project, Ministry of Health & Welfare, Republic of Korea (grant no. A102065-1011-1070100) | The authors have no conflicts of interest | 8 |

Abbreviations: GDM, gestational diabetes mellitus; OR, odds ratio; RR, risk ratio; NOS, Newcastle–Ottawa Scale.

Source of pre-pregnancy weight: self-reported or measured.

GDM diagnostic criteria: as reported in each original study.

Adjusted variables: covariates in each original model.

Funding and conflicts: extracted from each article; “Not reported” indicates unavailable; no conflicts declared in any study.

Quality: NOS score (maximum 9).

Note:Chen et al. (2025) originally used the Chinese Nutrition Society standard (<0 kg, 0–2 kg, >2 kg), which was harmonized to <0.5 kg, 0.5–2 kg, and >2 kg for this meta-analysis
