# Supplementary material for: Early pregnancy weight gain in pregnant women and the risk of gestational diabetes: a dose–response meta-analysis
Source: Front Public Health. 2026 Oct 29;14:1933184. doi: 10.3389/fpubh.2026.1933184 (PMC13754856; doi:10.3389/fpubh.2026.1933184)
Supplement: Supplementary file 2 [file Table_2.docx]

Table S2 Quality assessment of included cohort studies using the Newcastle‑Ottawa Scale (NOS)

| Representativeness of exposed cohort | Selection of non-exposed cohort | Ascertainment of exposure | Outcome not present at start | Comparability of cohorts | Assessment of outcome | Adequacy of follow-up (duration) | Adequacy of follow-up (completeness) | Total score |
| --- | --- | --- | --- | --- | --- | --- | --- | --- |
| 1 | 1 | 1 | 1 | 1 | 1 | 1 | 0 | 7 |
| 1 | 1 | 1 | 1 | 1 | 1 | 1 | 1 | 8 |
| 1 | 1 | 1 | 1 | 1 | 1 | 1 | 1 | 8 |
| 1 | 1 | 1 | 1 | 2 | 1 | 1 | 1 | 9 |
| 1 | 1 | 1 | 1 | 1 | 1 | 1 | 1 | 8 |
| 1 | 1 | 1 | 1 | 1 | 1 | 1 | 0 | 7 |
| 1 | 1 | 1 | 1 | 0 | 1 | 1 | 0 | 6 |
| 1 | 1 | 1 | 1 | 1 | 1 | 1 | 0 | 7 |
| 1 | 1 | 1 | 1 | 1 | 1 | 1 | 1 | 8 |
